# Bioluminescent Dinoflagellates as a Bioassay for Toxicity Assessment

**DOI:** 10.3390/ijms232113012

**Published:** 2022-10-27

**Authors:** Luíza S. Perin, Gabriela V. Moraes, Gabriela A. Galeazzo, Anderson G. Oliveira

**Affiliations:** 1 Department of Physical, Chemical and Geological Oceanography, Oceanographic Institute, University of São Paulo, São Paulo 05508-120, Brazil; 2 Department of Chemistry and Biochemistry, Yeshiva University, 245 Lexington Avenue, New York, NY 10016, USA

**Keywords:** circadian cycle, scintillons, biotechnological applications, ecotoxicological assays

## Abstract

Dinoflagellates bioluminescence mechanism depends upon a luciferin–luciferase reaction that promotes blue light emission (480 nm) in specialized luminogenic organelles called scintillons. The scintillons contain luciferin, luciferase and, in some cases, a luciferin-binding protein (LBP), which prevents luciferin from non-enzymatic oxidation in vivo. Even though dinoflagellate bioluminescence has been studied since the 1950s, there is still a lack of mechanistic understanding on whether the light emission process involves a peroxidic intermediate or not. Still, bioassays employing luminous dinoflagellates, usually from *Gonyaulax* or *Pyrocystis* genus, can be used to assess the toxicity of metals or organic compounds. In these dinoflagellates, the response to toxicity is observed as a change in luminescence, which is linked to cellular respiration. As a result, these changes can be used to calculate a percentage of light inhibition that correlates directly with toxicity. This current approach, which lies in between fast bacterial assays and more complex toxicity tests involving vertebrates and invertebrates, can provide a valuable tool for detecting certain pollutants, e.g., metals, in marine sediment and seawater. Thus, the present review focuses on how the dinoflagellates bioluminescence can be applied to evaluate the risks caused by contaminants in the marine environment.

## 1. Introduction

Bioluminescence can be defined as the production of visible light by living organisms [1] and it has been identified in many groups along the tree of life, such as insects, bacteria, cnidarians, ctenophores, squids, fishes, dinoflagellates, achieving the amount of 700 bioluminescent genera within 16 phyla [1,2,3]. Among these, luminescent marine clades are the most common, especially at depths where light does not reach [4], in comparison to the ocean surface where only 1–2% of the organisms are estimated to be luminescent [5].

Chemically, bioluminescence is produced by the oxidation of the molecule generally called luciferin by an enzyme (generally named luciferase), resulting in photons of visible light [5]. In some organisms, the bioluminescence emission is regulated by a circadian cycle, in other words, it may only be activated due to a biological cycle of 24 h usually synchronized with nycthemeral rhythms (day and night) [6].

An example of luminescent organisms that have a circadian cycle is the dinoflagellates, unicellular eukaryotes that can be photosynthetic and heterotrophic [7]. They are part of the plankton in marine and freshwater environments and are of major ecological importance, conducting processes such as photosynthesis, ecto- or endoparasite, and toxin production [8,9]. Therefore, dinoflagellates play an important ecological role by being critical food for filter-feeding organisms as well as for their algal blooms which may have negative effects with major environmental and human health impacts [10,11]. 

According to the latest data from AlgaeBase, there are 3711 species in Dinoflagellata superclass [12], among which only 68 have been classified as bioluminescent [7]. Dinoflagellates are also well known for causing the common sparkling water effect at night [11]. For example, the dinoflagellate species *Pyrodinium bahamense* has been identified as responsible for the ‘bioluminescent bays’ of Jamaica [12] and Puerto Rico [13]. The dinoflagellate *Noctiluca scintillans* is considered a major source of bioluminescence in many regions including the Sea of Cortez, Indian Ocean and Arabian Sea [14,15].

Even though quite remarkable by the sparkling water, the molecular mechanism of light emission in dinoflagellates is still not completely understood. Bioluminescence in dinoflagellates depends on a luciferin–luciferase reaction that promotes the light emission in flashes of intense blue light in specialized luminogenic organelles called scintillons. Light is primarily produced in response to mechanical stimulation [16], for example upon contact with a predator or by breaking waves [17]. However, whether the light emission process in dinoflagellates involves or not a peroxidic intermediate, and which is the real chemical nature of the oxidation product of this reaction (oxyluciferin) remains to be determined [1]. That should not be a reason to not apply this system as a biotechnological tool, though. Currently, even newly isolated bioluminescent systems are already being applied in several areas, such as biomedicine, biotechnology, and environmental sciences [18,19]. 

When it comes to environmental applications based on luminous bioassays, bioluminescent bacteria are the most common organism used. Low cost and quick response to contaminants are the main advantages of the use of these microorganisms [20]. Nonetheless, it is important to emphasize that bioassays that employ organisms of different trophic levels, should always be considered. In fact, the use of bioluminescent dinoflagellates as pollutants bioindicators can be considered a valuable tool, given the efficiency and low cost of carrying out such tests [21,22,23,24,25,26,27]. Furthermore, as they are eukaryotic cells, they can be considered better toxicity models for humans when compared to assays using bacteria [18]. Moreover, dinoflagellates constitute an important part of marine phytoplankton as primary producers, and their distribution and growth depend on environmental factors such as incidence of light, temperature, and nutrient concentration, which are ecologically relevant parameters for developing bioassay tools based on luminous dinoflagellates [28,29].

Thus, this review aims to revisit the biochemical aspects of light emission in dinoflagellates as well as to present environmental applications over the last decades using bioluminescent dinoflagellates. It is necessary to demonstrate the importance and potential of using these organisms as a tool for assessing environmental contaminants, especially when considering the main scenario of pollution growth and climate change.

## 2. Dinoflagellate Bioluminescence

### 2.1. Early Accounts

Dinoflagellates are single-celled eukaryotic organisms, which make up the group popularly known as microalgae. They are present in the most diverse aquatic environments, from coastal environments to the open ocean [30,31]. Dinoflagellates constitute an important part of the planktonic community of the oceans and freshwater environments, quite diverse in morphological and ecological terms. It is estimated that about 4500 species have been identified, approximately 2000 living species and 2500 fossil species [31,32], most of them inhabiting the marine environment. These organisms are primarily responsible for the luminescence on the sea surface. There are approximately 18 bioluminescent genera [33] of which *Noctiluca, Pyrocystis*, *Pyrodinium*, *Alexandrium*, *Gonyaulax* and *Protoperidinium* are the main representatives, which include 68 species of dinoflagellates with confirmed bioluminescence [34].

Dinoflagellates, for a long time, were described as the luminosity effect that could be seen when the surface of the water was shaken, without great details about how this light was produced and which organism was responsible for this emission [35]. This global phenomenon has been reported since at least 500 BC, but little attention has been given to understanding this phenomenon until the middle of the 19th century [35,36,37,38]. During the second half of the 18th century, the use of microscopes allowed the observation of small organisms, while analyzing sea water samples, and among them, the dinoflagellates. These were the first indications that the “phosphorescence” of the sea was caused by the presence of these small organisms in the water [39].

The dinoflagellate *N. scintillans* was first described by Macartney (1810) [34], who gave it the nomenclature *Medusa scintillans*. The stimulation of light emission in these organisms by adding an acidic solution was performed by Ehrenberg in 1834 [35]. Although studies on bioluminescence in this group were initiated in the 19th century, much of what is known today is mainly due to J. W. Hastings, who devoted several decades describing the process of light emission in these organisms [40]. Most of these descriptions were made using the species *Lingulodinium polyedrum* (formerly called *G. polyedra*), a specie usually responsible for red tides and bioluminescent blooms phenomena all over the world [8,38].

The chemical nature of the light emission process in dinoflagellates began to be elucidated only in the 1950s [38,39]. By the classic assay with hot and cold *L. polyedrum* cell extracts, Hastings and Sweeney demonstrated the requirement of three compounds for the light production reaction in addition to molecular oxygen: an enzyme (luciferase), a stimulant factor present in the boiled extract (later identified as luciferin), and salt, suggesting a luciferin-luciferase type reaction in the cell-free extracts of *L. polyedrum* [39,41]. A few decades ago, partial purification of dinoflagellate luciferin and luciferase were carried out for the first time by Bode and Hastings, in 1963 [42]. The authors were also able to show that the in vitro and in vivo bioluminescence emission spectra were remarkably similar. Additionally, during the chromatographic steps, the discovery of a novel luminescent particulate crystal-like structure was essential to understanding the process of light emission in dinoflagellates. The isolated particles, later named scintillons, could produce a flash of light by lowering the pH in the presence of oxygen, with the optimum luminescence within a pH of 5.7 [43]. Through sucrose density gradient experiments [44], a correlation between the light emission and the presence of scintillons was verified.

Later in the same decade, Fogel and Hastings [45] showed that scintillons were organelles that contain luciferin and proteins. One protein being a luciferase and, in some cases, a luciferin-binding protein (LBP) which is highly pH dependent, so the luciferin is released when the pH gets shifted from 8 to 6 [46]. The structure of dinoflagellate luciferin was only described in 1989, by Nakamura and colleagues [47], who showed luciferin molecule seems to be derived from chlorophyll (an open chain tetrapyrrole). *Pyrocystis lunula* was used as source of material for luciferin isolation since *P. lunula* produces greater amount of luciferin when compared to other dinoflagellate species. The crystal structure of luciferase was only elucidated in 2005 by Schultz and colleagues [48].

### 2.2. Overview of Biochemical Research

Bioluminescence in dinoflagellates is a typical luciferin–luciferase reaction in which light emission occurs in flashes of a few seconds (approximately 0.1 s) inside the scintillons, usually initiated by a mechanical disturbance. In these organelles are found luciferase and luciferin molecules, as well as the LBP protein, in a large part of this group. Species in this group are known to share a homologous type of luciferase, while LBP undergoes structural and genetic sequence variations [49]. 

**Figure 1:**
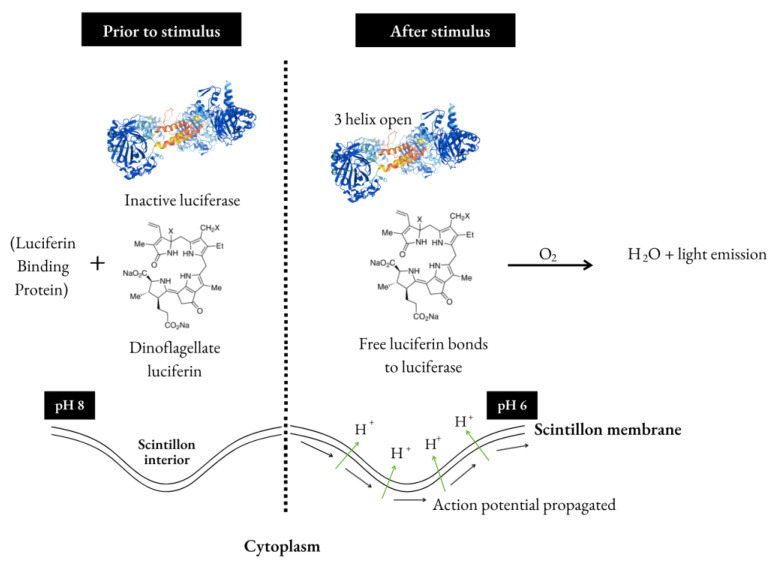
Schematic process for dinoflagellates bioluminescence in the scintillons.

The luciferin molecule present in the bioluminescent system of dinoflagellates, as mentioned, is an open chain tetrapyrrole, whose structure is similar to the chlorophyll molecule [47]. Although its structure has only been characterized in the species *P. lunula*, it is believed that this molecule is present in all species of bioluminescent dinoflagellates [38], being probably synthesized by photosynthetic species and acquired through the diet by heterotrophic species. Topalov [50] proposed in 2001 that the luciferin present in this group is possibly a product of chlorophyll degradation by photo-oxidation, however, this theory has only been shown to be valid for the species *P. lunula*. Yet, several authors have shown cross-reactivity between the dinoflagellate and krill bioluminescent systems [51]. This interesting feature can be explained by the structural resemblance of the dinoflagellate luciferin and krill luciferin (Figure 1). 

However, photosynthetic species are not the only ones that are bioluminescent. The luminescent heterotroph *Noctiluca scintillans* has a luciferin very similar to chlorophyll c, presumably obtained from its prey [52]. Nevertheless, a recent study on another heterotrophic dinoflagellate, *Protoperidinium crassipes* showed that even after one year being cultivated on rice flour, bioluminescence intensity persisted, showing that luciferin could not have been synthesized from chlorophyll, but produced endogenously [53]. Additionally, another hypothesis assumed that luciferin originated from chlorophyll photo-oxidation [50], but it is only valid for *P. lunula*, which maintains its luciferin throughout the light-dark cycle. On the other hand, fluorescent luciferin in *L. polyedrum* only appears during the dark phase [54], a period in which photo-oxidation is not possible [49], thus, its formation cannot be explained by the photo-oxidation mechanism. Therefore, there must be more than one mechanism responsible for luciferin production, even in photosynthetic species with closely related luciferases [36].

The light emission process in dinoflagellates is totally dependent on changes in the pH of the reactional medium. Scintillons are small organelles projected into the vacuole of the cell (acidic pH) and are connected to the cytoplasm (neutral pH) by a small channel [55]. At neutral pH, LBP is bound to luciferin, protecting it from non-enzymatic oxidation, while the enzymes (luciferases) are inactive [45]. The free form of luciferin is very unstable at neutral pH, but when bound to LBP, it becomes an extremely stable molecule [40]. Once the medium is acidified, the LBP protein releases the luciferin molecule and becomes inactive, making the substrate available to be oxidized by the now active luciferase, resulting in the formation of an oxyluciferin and a short, intense flash of light in the stain. In addition to rapid and intense emission, light can also be emitted gradually, reaching peak intensity, and returning to zero [49].

As mentioned, light emission in these organisms is a response to a mechanical disturbance, for example, caused by hydrodynamic turbulence, such as agitation and breaking waves. Once the cell membrane is subjected to mechanical stress, there is a propagation of a signal through an increase in the concentration of calcium (Ca^2+^) in the cytoplasm [56], causing the depolarization of the vacuole membrane and the associated scintillons generating an action potential. This process results in the activation of voltage-dependent ion channels [45,57], which allows a flow of protons from the outside (vacuole) to the inside of the scintillons, causing the pH change necessary for the chemical reaction of bioluminescence to occur. Some authors also point to the possible presence of an enzyme that returns H^+^ ions to the vacuole, reestablishing the pH inside the organelles [55]. In fact, it is known that a part of these protons is used in the formation of H_2_O molecules, also as a result of the bioluminescence reaction [36], but the mechanism by which this reestablishment takes place is still unclear.

Several behaviors among dinoflagellates, including the emission of light, are guided by a 24-h biological cycle known as the circadian cycle. It is known that throughout a cycle, the number of scintillons present in the cell of dinoflagellates varies, being more abundant during the dark period (night) and smaller during the light period (day) [58]. In some species, such as *L. polyedrum*, the entire bioluminescent system (luciferases, luciferins, and LBP) is consumed and synthesized at each cycle. Less than 10% of the scintillons synthesized overnight remain in the cell during the day [38]. In some species, such as *P. lunula*, during the light period, the scintillons migrate towards the cell nucleus and return to the cell wall during the dark period [38]. When the light emission is more intense, the concentration of luciferin and luciferase in the system is kept nearly constant. In addition to the circadian cycle, many species have their bioluminescence regulated by photoinhibition. Photoinhibition is believed to be an evolutionary adaptation so that light emission only occurs when it can be noticed (usually during the dark period), avoiding unnecessary energy expenditure. Some heterotrophic species of dinoflagellates lack the circadian cycle, with luminescence regulated only by photoinhibition [36]. Most of the studies related to the biochemistry of bioluminescence in dinoflagellates such as the presence of the LBP protein linked to luciferin, the circadian cycle, and the presence of specialized organelles were carried out to the use of the species *L. polyedrum* as a model.

The *G. polyedra* luciferase gene was first cloned in 1994 [59], then crystalized at the beginning of the 21st century [48] when it was found to be present in the same organization in other six dinoflagellates species [60], even though it is a completely novel structure when compared to other luciferases. Therefore, the dinoflagellates bioluminescence system is unique from both cellular and molecular perspectives [36]. The luciferase has three contiguous domains with very similar amino acid sequences, each 377 amino acids long, preceded by an N-terminal sequence [38]. Each of these domains alone is catalytically active, and each domain also shows the same pH dependency as the full-length enzyme [38].

With the acidification, the LBP (the species that contains it) [54,61,62,63] releases the luciferin (LH2) while the luciferase three-helix structure changes allowing LH2 to bind with the luciferase (Figure 1). When bonded, luciferin is oxidized by oxyluciferin with the participation of oxygen [11]. This process has been shown for the photosynthetic dinoflagellate *L. polyedrum* and it is suggested to represent most cases of bioluminescent photosynthetic dinoflagellates [48].

As well as not being universal to all dinoflagellates, LBP molecular studies have demonstrated a high variation in their sequences, showing a highly diverse gene family within the genome [64,65], for example in *L. polyedrum* [45,64], *Noctiluca scintillans* [66], *Alexandrium* spp. [67,68], and *P. lunula* [49]. The LBP has also been found in the genera *Gonyaulax*, *Ceratocorys*, and *Protoceratium* [69] (Table 1) as well as the genus *Pyrocystis* which has been recently added to the list of genera containing LBP [62,63,69]. On the other hand, the genera *Ceratium*, *Fragilidium*, and *Protoperidinium* lack the luciferin binding-protein [69]. Emerging information shows substantial evidence that LBP is an integral component of the standard molecular bioluminescence system in dinoflagellates [36,49,69].

When it comes to studying LBP, *L. polyedrum* is the model organism for it [64,70,71], in which LBP was found to be very abundant (up to 1% of the total proteome) [70]. It is believed that the region responsible for mediating the interaction between luciferase and luciferin binding protein is the LCF/LBP N-terminal domain [69]. 

Despite discoveries regarding LBP and its non-universal presence, the light production mechanism remains incomplete [72]. Unlike firefly bioluminescence, the reaction catalyzed by dinoflagellate luciferase (LCF) does not follow through with the formation of carbon dioxide; thus, LCF is likely to utilize a novel bioluminescence mechanism.

This mechanism is believed to be involved with a G-protein, a guanine nucleotide-binding protein [72], even though it remains unknown. G-proteins are a family of proteins responsible for molecular switches inside cells, such as signal transmitting from the exterior to a cell [84], being regulated by GTP (guanosine triphosphate) and guanosine diphosphate (GDP). When bound to GTP, G-proteins are active and when bound to GDP, are inactive [64].

Chen [84] studied in 2007 how G-proteins could be involved in the dinoflagellate *L. polyedrum* bioluminescence. The authors saw a strong inhibition of bioluminescence response by GDPβS, a G-protein inhibitor, thus, G-proteins must play a main role in the dinoflagellate bioluminescent system.

## 3. Bioluminescence as a Tool for Investigating Environmental Pollution

During the last decades, the molecular understanding of the light emission process by bioluminescent reactions has enabled its use in several biotechnological applications. Luminogenic substances and bioassays using bioluminescent organisms have been employed in several areas, allowing identification of tumors and infections, studies of gene expression and protein-protein interactions, as well as environmental sciences (toxicity bioassays/biosensors) [18].

The use of bioluminescence bioassays related to the marine environmental area is still an emerging field, however, its development is extremely important given the necessity to create new effective tools that contribute to the investigation and understanding of the marine pollution effects [85]. In fact, some pollutants are capable of bioaccumulating and/or biomagnifying at different trophic levels [86,87], such as Cu^2+^ and Cd^2+^, and therefore can impact different ecosystems and consequently affect the services they provide.

Bioassays can be defined as the use of living organisms to assess physiological or behavioral changes when they are exposed to toxic components [18,87]. Bioluminescent bioassays using bacteria and dinoflagellates proved to be a fast and reliable alternative for investigating the toxic potential of organic and inorganic pollutants in seawater, particularly when bioassays based on luminous organisms are compared to other common environmental tests based on the lethality of fish and aquatic invertebrates, or the effects on sea urchin fertilization and mussel larval development [18,21,22,23,24,25,26,27,88,89]. 

The use of bioluminescent bacteria in bioassays employs naturally bioluminescent bacteria (NBB) such as *Photobacterium fischeri*, formerly *Vibrio fischeri* [90], *Vibrio harvey*, *Photobacterium leiognathid*, and *Pseudomonas fluorescens*, or bacteria genetically modified (GMB) to emit light [18]. All these species of bioluminescent bacteria can be exposed to different sources of contaminated samples, including surface water, groundwater, wastewater, leachate, extracts of organic and aqueous sediments, and in short (acute) and long-term (chronic) tests [18,20].

The assessment of contaminants toxicity using NBB is done by registering the reduction in bioluminescence emission. Changes in bioluminescence are easily quantifiable endpoints, and there are various toxicity tests using light emission, for example, the Microtox^®^ test, which uses bioluminescent bacteria to detect toxic substances in different substrates. Thus, in these bacteria, the response to toxicity is observed as a change in luminescence, which is linked to cellular respiration. As a result, these changes can be used to calculate a percentage of light inhibition in the NBB that correlates directly with toxicity, allowing rapid and extremely sensitive responses to the presence of contaminants [18,20,91].

On the other hand, assays using GMB allow for a specific response to substances of interest, i.e., the genetic modification is made through the addition of a bioluminescent protein gene, which can be obtained from bacteria, fireflies, or *Aequorea victoria* jellyfish. Then, the luminescent protein is coupled to specific promoters related to target compounds, which can cause either the inhibition or the emission of light when the substance is present in the environment, depending on the desired response [18,92]. For example, an arsenic biosensor using *Escherichia coli* was developed using the construction of a specific reporter gene for this element. The authors fused the arsenic-binding regulatory protein gene, *ars*R (and a small portion of *ars*D), and its promoter, to the *A. victoria* green fluorescent protein (GFP) structural gene, resulting in a bacterial strain that produces fluorescence in response to arsenic exposure [92].

However, despite the advantages presented here of using bioluminescent bacteria in environmental assays, it is important to mention that different bioassays using organisms of different trophic levels should be performed in order to obtain a better understanding of the impacts generated by a specific pollutant in the ecosystem [18].

### 3.1. Environmental Application of Bioluminescent Dinoflagellates

The use of organisms that belong to the base of the food chain, such as dinoflagellates, has proved to be an important tool to predict possible environmental impacts generated by pollution since they are one of the first groups to be affected by changes caused by pollutants. Moreover, changes in the dynamics of their communities can affect the higher trophic levels of the ecosystem [93,94].

Nevertheless, the use of bioluminescent dinoflagellates for toxicity assessment is still poorly explored. Hannan and collaborators [26] published one of the seminal papers about the use of bioluminescent dinoflagellates in bioassays. In the work, *P. lunula* was employed to detect the presence of trichothecenes, mycotoxins produced by fungi of the genus *Fusarium* and *Stachybotrys*, which are responsible for causing a fatal disease in human beings known as food toxic aleukia [26]. The authors observed a reduction in light emission by *P. lunula* with the increase in the concentration of some types of trichothecenes, demonstrating the potential application of these dinoflagellates for the identification of toxic agents [26]. Based on this work, other researchers have used bioluminescent dinoflagellates in environmental bioassays, which are shown in Table 2.

*P. lunula* is the most widely used bioluminescent dinoflagellate species in environmental bioassays, followed by *L. polyedrum*. Both species of dinoflagellates have been extensively studied and are important constituents of marine phytoplankton. They can be cultivated in the laboratory using either natural or artificial seawater enriched with nutrients, with salinity around 33 and 35 and temperatures between 20 and 22 °C. The cultures are kept in incubators/growth chambers under reversed cycles of 12 h of darkness and 12 h of light, to allow the tests to be carried out in the dark phase of the cultures.

Luminescent dinoflagellates bioassays can be based either on the inhibition or on the re-establishment of bioluminescence after the dinoflagellates are exposed to a toxic substance [21,22,23,24,25,26,27,88,89]. Both tests use aliquots from the original culture with cell concentrations between 100 to 300 cells/mL, using a methodology based on counting cells through hemocytometer-type chambers, such as the Sedgewick-Rafter. Then, the light is measured in a luminometer, by stimulating the cells using agitation or the addition of an acetic acid solution. Bioluminescence inhibition or its recovery is calculated as a percentage of the control emission value.

#### 3.1.1. Bioluminescence Inhibition Bioassays

Several studies involving estimated IC_50_ values and inhibitory effects of inorganic (Table S1) and organic pollutants (Table S2) on the bioluminescence dinoflagellates bioassays have been published in the last years [21,22,24,25,88,89]. Most of the inorganic contaminants studied in the reviewed bioassays (Table S1) were endogenous constituents found in groundwater and some of them are associated with unconventional extraction of oil and natural gas (As^3+^, Ba^2+^, Se^4+^ and Sr^2+^). These contaminants were able to induce a quick inhibitory response within a period of 4 h, however, they also presented some high IC_50_ values (between 40 to 5400 mg/L) (Table S1), demonstrating an elevated tolerance of *P. lunula* to these substances [25]. 

As for other metals, great sensitivity from the luminous dinoflagellates was observed (IC_50_ ranging from 0.01 mg/L to 7 mg/L) (Table S1) [21,22,24,89], showing greater or similar sensitivity to conventional toxicity tests, such as those using as an endpoint the inhibition of *Daphinia sp*. growth, the inhibition of the bacterium *P. fischeri* bioluminescence [21], the survival rates of *Mysidopsis bahia* shrimp and *Menidia beryllina* fish [22,24], as well as embryo-larval development of mussels (*Mytilus* sp.). The latter is a test considered to be quite sensitive to metals [24]. However, being less sensitivity to Cu^2+^, when compared to growing inhibition tests of the algae *Nitzschia closteriume* (IC_50_ = 18 µg/L) and *Entomoneis c.f punctulata* (IC_50_ = 13 µg/L) [89]. 

Tests for assessing the toxicity of NH_3_ showed great sensitivity (IC_50_ = 0.068 mg/L) when compared to tests performed with mussels’ embryos (IC_50_ = 0.12–0.23 mg/L) [24]. Yet, assays using NH_4_^+^, considered less toxic than NH_3_ to *P. lunula*, showed that at low concentrations (<4 mg/L) light emission was stimulated and NH_4_^+^ was more toxic at 25 °C, compared to tests performed at 21 °C, based on NOEC (no observed effect concentration) values (1.3 and 7.4 mg N/L, respectively) [89]. The results also indicated that the sensitivity of *P. lunula* to NH_4_^+^ was higher than those experiments using bacterium *P. fischeri* bioluminescence assays, while was similar to chronic toxicity tests in algae and invertebrates, which makes dinoflagellates bioassays useful for chronic testing as they provide similar results in shorter periods of time [89].

For organic contaminants (Table S2), Stauber and collaborators [89] showed that TBT (Tributyltin), a biocide in anti-fouling paint, is moderately toxic to *P. lunula* (IC_50_ = 0.226 mg/L). In fact, *P. lunula* cultures seem to be less sensitive to TBT when compared to other algae such as *N. closterium* (IC_50_ = 0.0049 mg/L) and *E.* cf. *puntulata* (IC_50_ = 0.200 mg/L) [92]. In addition, tests of exposition using Diuron (IC_50_ = 19 mg/L), another algaecide and herbicide used in antifouling paint, suggested that *P. lunula* has in general lower sensitivity to this family of compounds. This makes the luminous dinoflagellates require the use of concentrations greater than 10 mg/L to achieve light emission inhibition. For instance, these values are higher than the concentrations necessary to inhibit the growth of *N. closterium* and *E.* cf. *punctulata* (6 µg/L) and algal photosynthesis (10–170 µg/L) [92]. 

Also, some authors reported that assays with glutaraldehyde and HCl, commonly used in unconventional natural gas drilling, also showed excessive IC_50_ values (5,16 mg/L and 194 mg/L, respectively) (Table S2). However, hydraulic fracturing fluid is documented to contain up to 0.01% glutaraldehyde and 0.13% HCl by mass, respectively, corresponding to approximate concentrations of 100 and 1300 mg/L. Thus, bioassays based on *P. lunula* bioluminescence would be able to respond to environmental contamination events in a short period of time [25].

The inhibitory effect of mixtures of contaminants on the bioluminescence of *L. polyedrum* was evaluated as well. Tests were carried out by exposing the luminous dinoflagellates to drain effluents containing metals, petroleum hydrocarbons, and suspended solids collected in San Diego Bay [21,22]. In these tests, limited effects were observed on dinoflagellates light emission when low effluent concentrations were used. In contrast, high sensitivity was observed when levels greater than 4% of the effluent were used [21,22]. These results are comparable with Microtox^®^ detection [21].

Okamoto and collaborators tested four different metals (Cu^2+^, Hg^2+^, Pb^2+^ and Cd^2+^) against the dinoflagellate *L. polyedrum* (Table 2). The authors observed differences in light emission, with an increase in the frequency of light flashes after the exposure of the protists to the contaminants and minor changes in the period and amplitude of the bioluminescence rhythm, evidencing an impact generated by metals in the bioluminescence system of these algae [88].

Due to the ability to indicate contamination, commercial kits using tests that evaluate the inhibitory effect of the bioluminescence dinoflagellates, for example, QwikLite^TM^, are available on the market. This bioassay uses 3 mL aliquots, containing the dinoflagellates and the substance to be analyzed, and the emission of light is stimulated by agitation. The results can be obtained in approximately 2–3 h and can be conducted for up to 7 days (chronic tests) [20,21].

#### 3.1.2. Bioluminescence Re-Establishment Bioassays

In studies that analyzed the potential for restoring the bioluminescence in treatments with pollutants [23,27], *P. lunula* was used and a dose-dependent effect was observed on the dinoflagellate recovery capacity for all tested contaminants (Table 2) [27], with Cu^2+^ (IC_50_ = 0.96 µM/L) and Cd^2+^ (IC_50_ = 1.18 µM/L) having the greatest toxic effect [27]. Heimann and collaborators [27] also studied the bioluminescence recovery capacity of protists when they were placed for 72 h in an uncontaminated culture medium and kept under culture maintenance conditions after treatment with pollutants. The results showed that only cells exposed to Ni^2+^ and Pb^2+^ had irreversible bioluminescence inhibitory effects. Treatments with Cu^2+^, Cd^2+^, and organic pollutants were reversible, returning between 80–100% of bioluminescence after 72 h, which demonstrates an efficient capacity for detoxification of *P. lunula* to a wide variety of contaminants [27].

The effects of changes in pH, salinity, and temperature on the light restoration when dinoflagellates were exposed to Cu^2+^ and SDS, were analyzed in order to evaluate possible conditions that may occur in the natural environment [23]. The authors concluded that bioassays employing *P. lunula* do not need to be very restrictive to changes in these environmental variables, since small changes in pH, salinity and temperature do not significantly alter the potential of bioluminescence restoring, which makes it possible to extrapolate the results to other environmental conditions [23].

## 4. Final Considerations and Perspectives

Through the data collected and analyzed in this review, it is evident that bioassays employing bioluminescent dinoflagellates can be reliable tools to assist in the investigation of environmental pollution. Bioassays based on luminous dinoflagellates are of low cost, capable of presenting rapid results, and use a eukaryotic organism as a bioindicator, which is a better predictor of toxic effects in humans when compared to other conventional tests using bacteria [18]. In addition, dinoflagellates are primary producers, being able to provide an initial response to possible negative impacts on marine biota and ecosystem services provided by the ocean. 

However, despite the advantages of using bioluminescent dinoflagellates in environmental bioassays, further studies will be necessary to increase our molecular understanding of how these organisms emit light and, most importantly, how pollutants can affect this mechanism. For example, Okamoto and collaborators [88] suggest that the changes in the frequency of light flashes when *L. polyedrum* culture was exposed to metals are due to the ability of these elements to stimulate action potentials in the scintillons vascular membrane, triggering the emission of light. Since toxic metal ions are capable of damaging the membrane, causing its depolarization and acidifying the cytoplasm, Heimann and collaborators [27] propose a possible partial depletion of the bioluminescent system would occur, since no morphological or cell density changes were observed in relation to the control [27]. In this way, investigating the mechanism of light emission in dinoflagellates will open up new perspectives for both the analytics and the understanding of these cellular processes.

## Figures and Tables

**Table 1:** Compilation of dinoflagellates biological and luminescence data.

Dinoflagellate Species	Diameter	Feeding Mechanism	Luciferase Size (kDa)	Luciferin-Binding Protein
*Alexandrium* spp.	30–82 μm	Photosynthetic	137	Present
	[73]	[36]	[66]	[74]
*Ceratocorys horrida*	~70 μm	Photosynthetic	----	Present
	[75]	[75]		[69]
*Gonyaulax* spp.	24–75 μm	Photosynthetic	130	Present
	[76]	[52]	[77]	[78]
*Lingulodinium polyedrum*	37–53 μm	Photosynthetic	137	Present
	[79]	[36]	[78]	[78]
*Noctiluca scintillans*	0.2–2 mm	Heterotrophic	100	Present
	[80]	[49]	[52]	[52]
*Protoperidinium crassipes*	65–110 μm	Heterotrophic	----	Not present
	[53]	[81]		[69]
*Pyrocystis fusiformis*	370 μm	Photosynthetic)	~137	Not present
	[82]	[66]	[66]	[62]
*Pyrocystis lunula*	100–140 μm	Photosynthetic	~137	Present
	[83]	[49]	[78]	[70]

**Table 2:** Environmental bioassays performed with bioluminescent dinoflagellates.

Bioluminescent Dinoflagellate Used	Tested Substances	Test Type	Test Duration	References
*Pyrocystis lunula*	Trichthecenes	Inhibition of bioluminescence	2 h	[26]
*Lingulodinium polyedrum* and *Pyrocystis lunula*	Tributyltin chloride, copper, zinc, and storm drain effluent	Inhibition of bioluminescence	4 h to 11 days	[21]
*Lingulodinium polyedrum*	Copper, storm drain effluent, and polyaromatic cyclic hydrocarbons (PAHs)	Inhibition of bioluminescence	24 h, 48 h, 72 h and 96 h	[22]
*Lingulodinium polyedrum*	Copper, cadmium, lead, and mercury	Inhibition of bioluminescence	48 h, 96 h and 8 days	[88]
*Pyrocystis lunula*	Copper, cadmium, lead, nickel, Sodium dodecyl sulfate (SDS), phenol, and phenanthrene	Bioluminescence re-establishment	4 h	[27]
*Pyrocystis lunula*	Sodium dodecyl sulfate, (SDS) and copper	Bioluminescence re-establishment	4 h	[23]
*Lingulodinium polyedrum*, *Ceratocorys horrida* and *Pyrocystis noctiluca*	Copper, cadmium, lead, mercury, silver, zinc, chrome, and non-ionized ammonia	Inhibition of bioluminescence	24 h	[24]
*Pyrocystis lunula*	Tributyltin (TBT), copper, diuron, and ammonia	Inhibition of bioluminescence	24 h, 48 h, 96 h and 120 h	[89]
*Pyrocystis lunula*	Glutaraldehyde, hydrochloric acid (HCl), arsenic, selenium, barium, and strontium	Inhibition of bioluminescence	4 h, 6 h, 8, 12 h, 24 h, 48 h, 72 h and 98 h	[25]

## Supplementary Materials

The supporting information can be downloaded at: https://www.mdpi.com/article/10.3390/ijms232113012/s1.
