# Supplementary material for: Bioluminescent Dinoflagellates as a Bioassay for Toxicity Assessment
Source: Int J Mol Sci. 2022 Oct 27;23(21):13012. doi: 10.3390/ijms232113012 (PMC9656108; doi:10.3390/ijms232113012)
Supplement: Supplementary file 1 [file ijms-23-13012-s001.zip › ijms-1851067-supplementary.pdf]

Supplementary Materials

Table S1. IC50 values for the inorganic contaminants tested with bioluminescent dinoflagellates.

Substances	Hour	*Pyrocystis lunula*	*Lingulodinium polyedrum*	*Ceratocorys horrida*	*Pyrocystis noctiluca*	Reference
Copper	4	-	0.085 mg/L	-	-	[21]
	24	2 mg/L	0.07 mg/L	-	-	[21]
		-	0.09 mg/L	0.166 mg/L	0.185 mg/L	[24]
		0.128 mg/L	-	-	-	[92]
	48	3.127 mg/L	-	-	-	[92]
		3.2 mg/L	0.065 mg/L	-	-	[21]
	72	-	0.06 mg/L	-	-	[21]
	96	2.1 mg/L	-	-	-	[21]
Zinc	3	7 mg/L	-	-	-	[21]
	24	-	0.349 mg/L	0.349 mg/L	0.345 mg/L	[24]
	48	1.7 mg/L	-	-	-	[21]
Cadmium	24	-	0.843 mg/L	1.71 mg/L	1.13 mg/L	[24]
Lead	24	-	0.747 mg/L	2.06 mg/L	2.71 mg/L	[24]
Mercury	24	-	0.008 mg/L	0.021 mg/L	0.01 mg/L	[24]
Silver	24	-	0.006 mg/L	0.008 mg/L	0.038 mg/L	[24]
Chrome	24	-	1.83 mg/L	2.04 mg/L	1.94 mg/L	[24]

Substances	Hour	*Pyrocystis lunula*	*Lingulodinium polyedrum*	*Ceratocorys horrida*	*Pyrocystis noctiluca*	Reference
Arsenic	4	2.55 mg/L	-	-	-	[25]
	24	2.55 mg/L	-	-	-	[25]
	96	2.55 mg/L	-	-	-	[25]
Barium	4	40 mg/L	-	-	-	[25]
	24	68 mg/L	-	-	-	[25]
	96	70 mg/L	-	-	-	[25]
Selenium	4	200 mg/L	-	-	-	[25]
	24	307 mg/L	-	-	-	[25]
	96	359 mg/L	-	-	-	[25]
Strontium	4	5400 mg/L	-	-	-	[25]
	24	3200 mg/L	-	-	-	[25]
	96	5100 mg/L	-	-	-	[25]
Un-ionized ammonia	24	-	0.068 mg/L	0.142 mg/L	0.405 mg/L	[24]

**Substances**	**Hour**	***Pyrocystis lunula***	***Lingulodinium polyedrum***	***Ceratocorys horrida***	***Pyrocystis noctiluca***	**Reference**
Ammonia	24	10 mg/L	-	-	-	[92]
	48	9.2 mg/L	-	-	-	[92]
	120	9 mg/L	-	-	-	[92]

Table S2. IC_{50} values for the organic contaminants tested with bioluminescent dinoflagellates

**Substances**	**Hour**	***Pyrocystis lunula***	***Lingulodinium polyedrum***	***Ceratocorys horrida***	***Pyrocystis noctiluca***	**Reference**
Tributyltin Chloride (TBTCl)	120	0.0085 mg/L	-	-	-	[21]
	204	0.003 mg/L	-	-	-	[21]
Tributyltin (TBT)	24	0.226 mg/L	-	-	-	[92]
Diuron	24	19 mg/L	-	-	-	[92]
Glutaraldehyde	4	4.60 mg/L	-	-	-	[25]
	24	5.14 mg/L	-	-	-	[25]
	96	4.46 mg/L	-	-	-	[25]
HCL	4	136 mg/L	-	-	-	[25]
	24	194 mg/L	-	-	-	[25]
	96	274 mg/L	-	-	-	[25]
